# Cytotoxic effects of targeted agent alone or with chemotherapy in the treatment of adenoid cystic carcinoma: a preclinical study

**DOI:** 10.1038/s41598-022-14197-8

**Published:** 2022-06-15

**Authors:** Teresa Savarese, Andrea Abate, Ram Manohar Basnet, Luigi Lorini, Cristina Gurizzan, Michele Tomasoni, Davide Lombardi, Davide Tomasini, Daniela Zizioli, Maurizio Memo, Alfredo Berruti, Sara A. Bonini, Sandra Sigala, Paolo Bossi

**Affiliations:** 1 Section of Pharmacology, Department of Molecular and Translational Medicine, University of Brescia, Viale Europa 11, 25123 Brescia, Italy; 2 Section of Biotechnology, Department of Molecular and Translational Medicine, University of Brescia, Viale Europa 11, 25123 Brescia, Italy; 3 Medical Oncology Unit, Department of Medical and Surgical Specialties, Radiological Sciences, and Public Health, University of Brescia and ASST Spedali Civili, Piazzale Spedali Civili 1, 25123 Brescia, Italy; 4 Unit of Otorhinolaryngology-Head and Neck Surgery, ASST Spedali Civili di Brescia, Department of Medical and Surgical Specialties, Radiologic Sciences, and Public Health, University of Brescia, Piazzale Spedali Civili 1, 25123 Brescia, Italy; 5 Radiation Oncology Unit, Department of Medical and Surgical Specialties, Radiological Science and Public Health, ASST Spedali Civili of Brescia, University of Brescia, Piazzale Spedali Civili 1, 25123 Brescia, Italy

**Keywords:** Cancer, Cancer therapy, Head and neck cancer, Oral cancer

## Abstract

Adenoid cystic carcinoma (ACC) is a rare malignancy characterized by high incidence of relapse. When relapsing, ACC has an indolent but relentless behaviour, thus leading to a poor long-term prognosis. The treatment of choice of relapsing ACC remains surgery followed by radiotherapy, whenever feasible. Therapeutic weapons are limited to systemic drugs. The most widely used chemotherapy regimen is the combination of cisplatin and doxorubicin, however with low response rate and not long lasting; there is also a lack of alternatives for second line therapies in case of disease progression. Therefore, a more comprehensive strategy aimed at identifying at preclinical level the most promising drugs or combination is clearly needed. In this study, the cytotoxic effects of two standard chemotherapy drugs, cisplatin and doxorubicin, and of five targeted therapy-drugs was tested in vitro, on an h-TERT immortalized ACC cell line, and in vivo, on zebrafish embryos with ACC tumoral cell xenograft. Then, combinations of one standard chemotherapy drug plus one targeted therapy drug were also evaluated, in order to find the best treatment strategy for ACC. Data obtained demonstrated that both vorinostat and olaparib significantly increased the standard chemotherapy cytotoxic effects, suggesting new interesting therapeutic options for ACC.

## Introduction

Adenoid cystic carcinoma (ACC) is a rare malignancy arising mostly from salivary glands and from other sites, as trachea, bronchi, breast and skin, with an incidence of 4.5 cases per 100,000 individuals^1^. It represents 20% of malignant tumors of the major salivary glands and 58% of the minor salivary glands^2^. ACC is reported to relapse in till 50–60% of the cases, both locally and at distant sites^3–5^.

Generally, when relapsing, ACC has an indolent but relentless behaviour, thus leading to a poor long-term prognosis; 10-year OS ranges from 52 to 65% in several retrospective series^6, 7^.

Locoregional or distant recurrences represent a crucial part of the patient journey, as negatively impacting on prognosis and with higher risk of patient’s quality of life deterioration. The treatment of choice of relapsing ACC remains surgery followed by radiotherapy, whenever feasible. In case of absence of indications for locoregional treatments, with a patient experiencing symptomatic disease or at high risk of complications, therapeutic weapons are limited to systemic drugs.

The most widely used chemotherapy regimen is the combination of cisplatin and doxorubicin, however with response rate (RR) of 25% and not long lasting; there is also a lack of alternatives for second line therapies in case of disease progression^8^. Several target agents have been studied in ACC, mostly as single agents and in the absence of molecular selection. Recently, the most promising results have been obtained with multikinase inhibitor lenvatinib and with antiangiogenetic drugs as sorafenib and axitinib; however, these drugs reported a response rate of about 15% with a non-negligible burden of adverse events^9–12^. The most frequently reported molecular alteration, aside the MYB/MYB1 pathognomonic fusions, is the NOTCH-1 mutation^13^; in this regard, tailored targeted approaches have been studied and are currently ongoing in NOTCH-mutated ACC^14^. Overall, targeted agents seem to benefit a small quote of recurrent and/or metastatic (RM) ACC, at the price of drug-induced toxicities. Therefore, a more comprehensive strategy aimed at identifying at preclinical level the most promising drugs or combination is clearly needed.

The primary aim of our study is the evaluation in vitro and in vivo of new therapeutic strategies that can expand therapeutic possibilities available for patients with RM ACC.

## Methods

### Cell culture

The human ACC (hTERT) cell line, derived from a primary untreated and predominantly cribriform ACC of the tongue base immortalized using h-TERT transfection, was kindly given by Prof. Adel El-Naggar (Department of Pathology, The University of Texas, MD Anderson Cancer Center, Houston, TX, USA). Cells were maintained in standard medium, as previously reported^15^. ACC (hTERT) cells doubling time both at 37 °C and at 32 °C, 5% CO_2_ was calculated according to ATCC (American Type Culture Collection, Virginia, USA). Cells were authenticated by the GenePrint 10 System (Promega Italia, Milan, Italy), according to the protocols suggested by the manufacturer. Cells were used between passage 144 and 155 and periodically tested for mycoplasma.

### Cells treatment

ACC (hTERT) cells (15,000 cells/well) were plated in 24-well plates with complete medium. For the concentration–response curves, cells were exposed to the following drugs concentration: cisplatin (Selleckchem Chemicals, Houston, Texas, USA) (0.03–24 µM); doxorubicin (Selleckchem Chemicals) (0.001–1 µM); lenvatinib (MedChem Express, Monmouth Junction, New Jersey, USA) (0.05–2 µM); vorinostat (MedChem Express) (0.1–9 µM); everolimus (Selleckchem Chemicals) (0.5–250 nM); palbociclib (Selleckchem Chemicals) (0.03–9 uM); olaparib (Selleckchem Chemicals) (0.15–18 µM). According to cells doubling time, ACC (hTERT) cells were treated with the drugs for 4 days.

### Cell viability and cell proliferation assay

Cell viability was assessed by 3-(4,5-Dimethyl-2-thiazol)-2,5-diphenyl-2H-tetrazolium bromide (MTT) dye reduction assay according to the manufacturer protocol (Sigma Aldrich, Italia, Milan, Italy) and performed as previously described^16^. Cell proliferation rate was evaluated with TC20 automated cell counter (Bio-Rad, Segrate, Milan, Italy).

### Drug combination experiments

Drug combination experiments were performed to evaluate drug interactions on ACC cell viability, according to the Chou and Talaly method^17^. Cells were treated for 4 days with the following drugs, used alone or in combination: cisplatin (0.018–13.2 µM) and palbociclib (0.020–14.742 µM), doxorubicin (0.002–0.255 µM) and palbociclib (0.026–3 µM), cisplatin (0.187–21.3 µM) and vorinostat (0.064–7.26 µM), doxorubicin (0.002–0.255 µM) and vorinostat (0.14–16 µM), cisplatin (0.1–30.37 µM) and everolimus (0.41–157.46 nM), doxorubicin (0.002–0.255 µM) and everolimus (0.62–70.3 nM), cisplatin (0.75–25.66 µM) and olaparib (0.874–29.74 µM), doxorubicin (0.002–0.255 µM) and olaparib (0.23–26.6 µM), vorinostat (0.018–13.5 µM) and palbociclib (0.02–14.58 µM). Drug concentrations used in the combinations followed a fixed dilution ratio, as recommended for the most efficient data analysis^18^. Data were analysed using the CompuSyn software (ComboSyn inc. Paramus, NJ, USA) as previously described^16^.

### Collection of zebrafish eggs and fish maintenance

All zebrafish were handled according to national and international guidelines (EU Directive 2010/63/EU), following protocols approved by the local committee (Organismo Preposto al Benessere Animale (OPBA), Università degli Studi di Brescia, protocol no. 211B5.24) and authorized by the Ministry of Health (authorization no. 393/2017-PR). The experiments were complied with the ARRIVE guidelines (https://arriveguidelines.org). Healthy adult wild-type zebrafish (AB strain) were used for egg production. Fishes were maintained under standard laboratory conditions^19^, at 28 °C on a constant 14-h light/10-h dark cycle. Fish were fed thrice daily with a combination of granular dry food and fresh artemia (Special Diet Services, SDS Diets; LBS Biotech, Horley, UK). Collection of zebrafish eggs and maintenance of developing embryos has been performed as previously described^20^.

### Zebrafish single-drug treatment concentration

Embryos maintained in fish water plus PTU (1-phenyl-2-thiourea) were manually dechorionated at 48 h post-fertilization (hpf) and treated for 3 days with each single drug at these concentration range: cisplatin (25–50 µM), doxorubicin (5–20 µM), vorinostat (25–50 µM), palbociclib (5–10 µM), olaparib (25–100 µM), everolimus (1–5 µM). For each drug the toxicity was tested, and the final effective concentration was set for subsequent experiments.

### Tumor xenograft

ACC cells (1.25 × 10^6^ cells) were labelled with the vital red fluorescent dye CellTrackerTM CM-DiI (final concentration 0.66 ng/mL; Thermo Fisher Scientific, Milan, Italy), and resuspended in 25 µL of PBS for microinjections. Tumor xenograft was performed as previously described^20^. Drugs or solvents were directly added to the PTU-fish water and after 3 days of treatment (T3), pictures were taken. The tumour areas of vehicle- and drug-treated groups at T0 and T3 were analysed with Axio Zoom Fluorescent Microscope (Carl Zeiss AG, Oberkochen, Germany) and measured with Noldus DanioScopeTM software (Noldus information Technology). Data obtained were analysed by GraphPad Prism software version 6.01.

### Statistical analysis

Data analysis was performed using GraphPad Prism software version 5.02 (GraphPad Software, La Jolla, CA). Statistical analysis was carried out using one-way ANOVA analysis with a post hoc test (Bonferroni’s test) for multiple comparisons, considering p < 0.05 as threshold for statistical significance. Data are expressed as mean ± SEM of three independent experiments, unless otherwise specified. Cytotoxicity experiments were carried out at least three times, each point run in triplicate.

## Results

### Standard chemotherapy and target therapy drugs induced cytotoxicity in ACC cell line

ACC (hTERT) cell line doubling time was firstly evaluated (Fig. S1). Being the cells doubling time around 50 h, ACC cells were treated with the drugs for 4 days.

The cytotoxicity of the two standard chemotherapeutic agents (cisplatin and doxorubicin) and five target therapy drugs (everolimus, palbociclib, olaparib, vorinostat and lenvatinib) were tested on ACC (hTERT) cell line.

**Figure 1:**
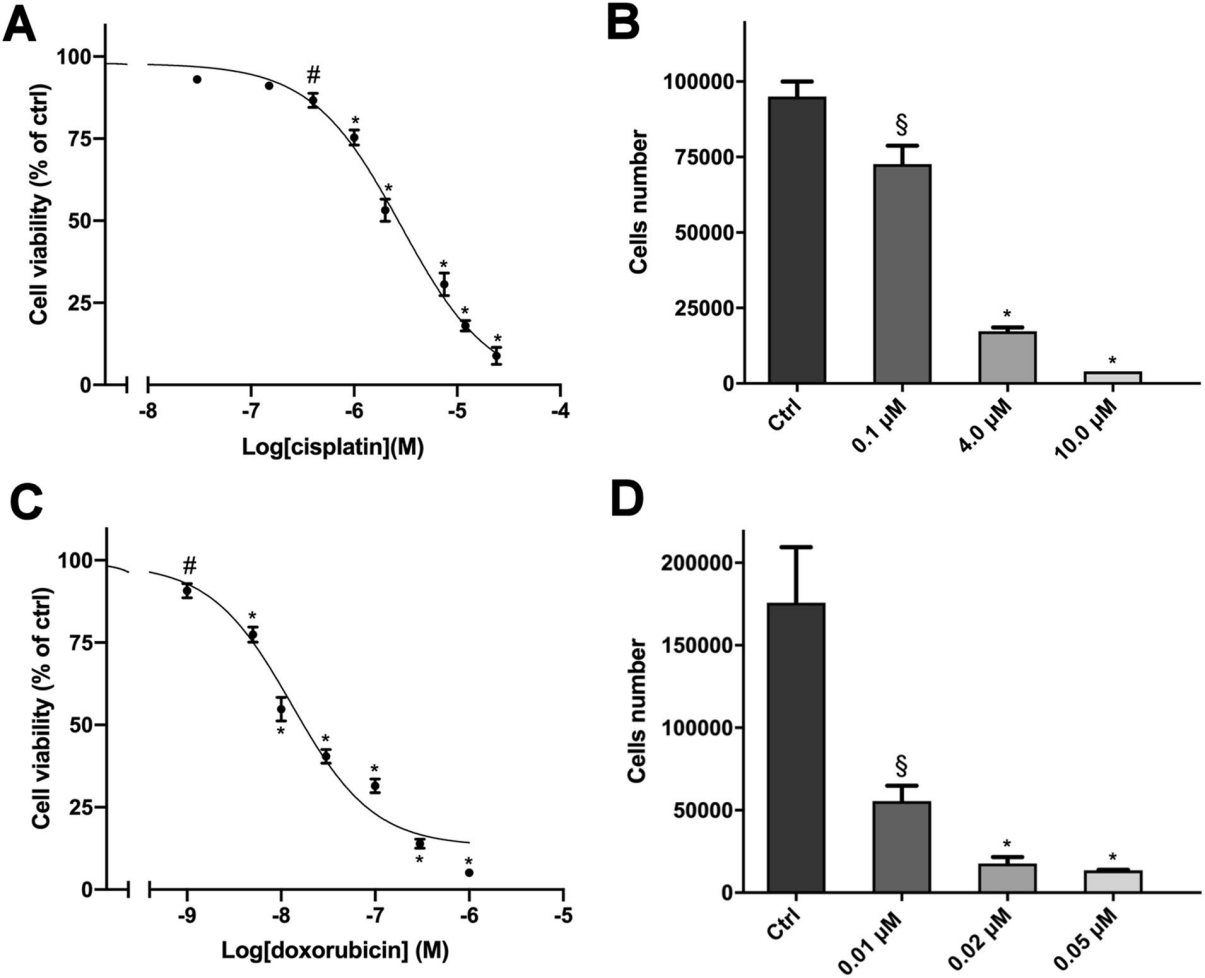
Cytotoxic effect of cisplatin and doxorubicin in hTERT cells. (**A**) Concentration–response curve of cisplatin-induced inhibition of cell viability. Cells were treated with increasing concentrations of cisplatin (0.03–24 µM) for 4 days. (**B**) Effect of cisplatin on cell proliferation. Cells were treated for 4 days with three concentrations of cisplatin. (**C**) Concentration–response curve of doxorubicin-induced inhibition of cell viability. Cells were treated with increasing concentrations of doxorubicin (0.001–1 µM) for 4 days. (**D**) Effect of doxorubicin on cell proliferation. Cells were treated for 4 days with three concentrations of doxorubicin. Cell viability was evaluated by MTT assay while cells proliferation was assessed after cells count with trypan blue exclusion. Data are shown as mean ± SEM [*p < 0.0001; ^#^p < 0.01; ^§^p < 0.05].

Exposure of cells to increasing concentrations of cisplatin and doxorubicin for 4 days led to a concentration-dependent reduction of cell viability, analysed by MTT assay. Sigmoidal concentration–response function was applied to calculate the IC_50_ value, that was, respectively, 3 μM (95% confidence interval (CI): 2.3–3.5 μM) and 0.013 μM (95% CI 0.01–0.017 μM) for cisplatin and doxorubicin (Fig. 1A,C). The standard chemotherapy drugs were highly active in inducing cytotoxicity, as their efficacy reached over the 90% at the highest concentration tested.

Also, a cell count was performed in order to evaluate the effect on the cell proliferation rate. As reported in Fig. 1B,D, cell count confirmed data obtained in the MTT viability test.

**Figure 2:**
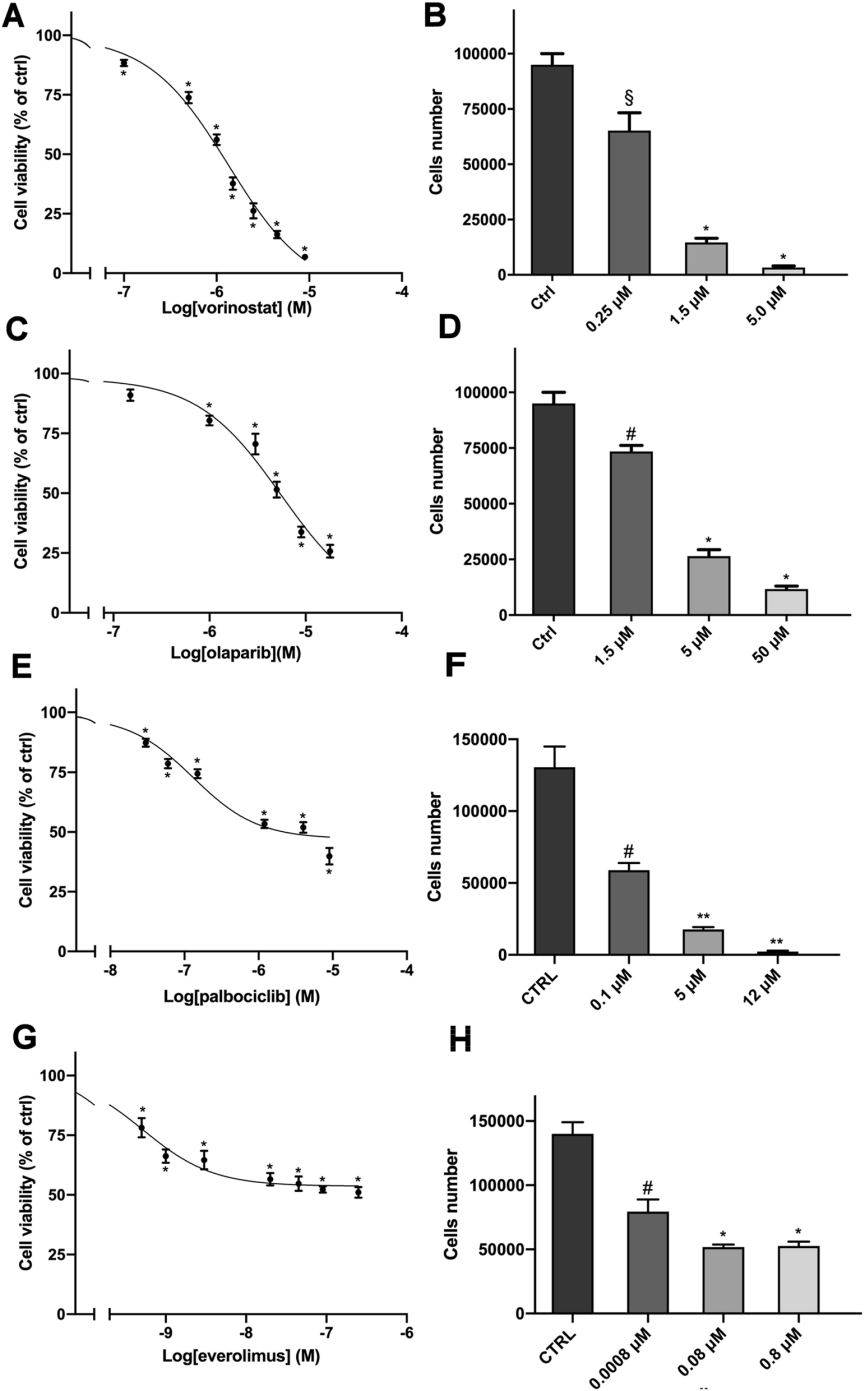
Cytotoxic effect of target therapy drugs in hTERT cells. (**A,C,E,G**) Concentration–response curves of vorinostat-, olaparib-, palbociclib- and everolimus-induced inhibition of cell viability. Cells were treated with increasing concentrations of vorinostat (0.1–9 µM) or olaparib (0.15–18 µM) or palbociclib (0.03–9 µM) or everolimus (0.5–250 nM) for 4 days. (**B,D,F,H**) Effect of vorinostat, olaparib, palbociclib and everolimus respectively, on cell proliferation. Cells were treated for 4 days with three concentrations of each drug. Cell viability was evaluated by MTT assay while cells proliferation was assessed after cells count with trypan blue exclusion. Data are shown as mean ± SEM [*p < 0.0001; ** p<0.001; ^#^p < 0.01; ^§^p < 0.05].

We then evaluated the effect of ACC potentially effective target therapy drugs. Among the drugs tested, the four drugs listed below resulted to be effective in reducing cell viability, considering drug concentrations compatible with clinical practice: vorinostat, olaparib, palbociclib and everolimus (Fig. 2A,C,E,G). The IC_50_ values were: vorinostat 1.3 µM (95% CI 1.1–1.6 μM), olaparib 5.5 µM (95% CI 3.8–8.1 μM), palbociclib 0.13 µM (95% CI 0.09–0.2 μM), everolimus 0.5 nM (95% CI 0.3–0.8 nM). Drug effects on cell proliferation were also evaluated, as reported in Fig. 2B,D,F,H. Detailed data on drugs potency and efficacy are reported in Table 1. Lenvatinib was as well tested for cytotoxic effects on ACC (hTERT) cells, but its efficacy was very poor, remaining far above the 50% value of cell viability even at high drug concentrations (Fig. S2).

**Table 1:** Drugs potency and efficacy.

Drugs	IC_50_ (95% C.I.)	% cell mortality (± SEM) at max concentration (M)
Cisplatin	3 μM (2.3 to 3.5 μM)	91.15% (± 2.6) at 24 μM
Doxorubicin	0.013 μM (0.01 to 0.017 μM)	94.91% (± 0.77) at 1 μM
Vorinostat	1.3 μM (1.1 to 1.6 μM)	96.28% (± 0.47) at 13.5 μM
Olaparib	5.5 μM (3.8 to 8.1 μM)	74.26% (± 2.69) at 18 μM
Palbociclib	0.13 μM (0.09 to 0.2 μM)	60.13% (± 3.4) at 9 μM
Everolimus	0.5 nM (0.3 to 0.8 nM)	48.96% (± 2.23) at 250 nM

### In vivo effects of both standard chemotherapy and target therapy drugs

**Figure 3:**
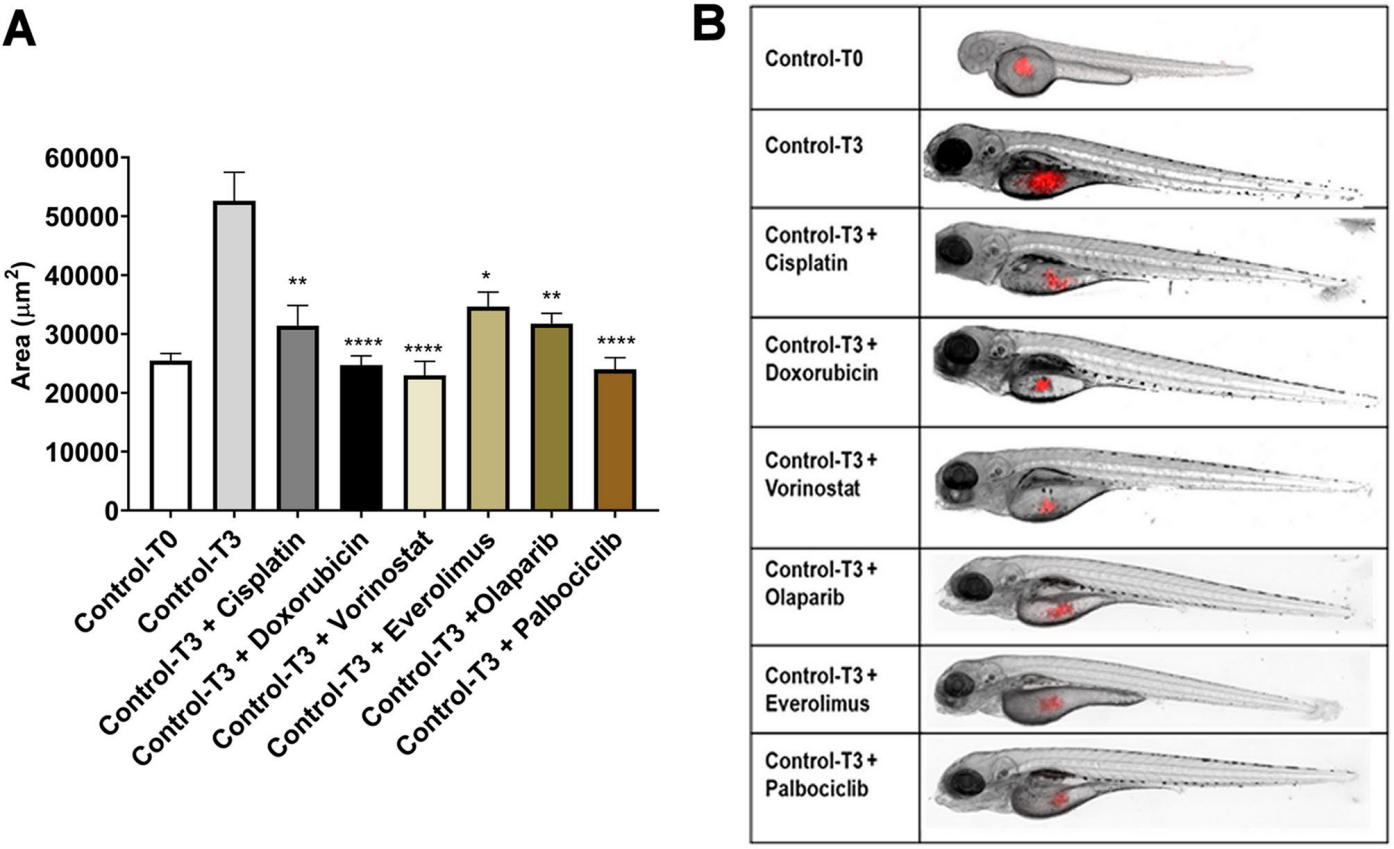
Chemotherapy drugs and target therapy drugs reduced the tumor xenograft area of hTERT cells. (**A**) The tumor area of T0 and T3 drug-treated and solvent-treated groups was measured with Noldus DanioScopeTM software. (**B**) A representative image is shown. Control-T0, time point at injection (control embryos at 48 hpf); Control-T3, time point 3 days later in fish water with solvent alone (untreated embryos at 120 hpf); Control-T3+ drugs, time point 3 days later in fish water with drugs (treated embryos at 120 hpf). The experiments were performed twice and each group was representative of 25 embryos. Data are shown as mean ± SEM [****p < 0.0001; **p < 0.01; *p < 0.05].

The cytotoxic effects observed in vitro was then tested in vivo in the experimental model of cell xenograft on zebrafish embryos. As zebrafish embryos were maintained at the temperature of 32 °C, ACC cell behavior was studied in terms of viability and doubling time at 32 °C and results indicated that this cell line was able to growth and duplicate at this temperature (Fig. S3). The ACC cells were injected in zebrafish embryos and the tumor area was evaluated at different times. In particular, each single drug or solvent were directly added to the fish water and after 3 days of treatment the tumor areas of vehicle- and drug-treated groups at T0 (2 h after treatment) and T3 (3 days of treatment) were measured (Fig. 3B).

As reported in Fig. 3A, ACC tumor grew very fast in the injected area of the yolk sac, approximately doubling its area in 72 h (mean area (μm^2^) ± SEM: 29.11 ± 0.9 × 10^3^ μm^2^ and 52.6 ± 4.9 × 10^3^ μm^2^ at T0 and T3, respectively). All the drugs tested significantly reduced the tumor area compared to T3-vehicle. In particular, doxorubicin, vorinostat and palbociclib exposure reduced the tumor area at levels comparable to the T0 group (mean area (μm^2^) ± SEM: 24.7 ± 1.6 × 10^3^ μm^2^; 23.0 ± 2.3 × 10^3^ μm^2^ and 24.0 ± 2.0 × 10^3^ μm^2^, respectively). Cisplatin, everolimus and olaparib treatment, although less effective in reducing tumor area compared to the other drugs tested, led to a significant difference as compared to the T3-vehicle group (mean area (μm^2^) ± SEM: 31.4 ± 3.4 × 10^3^ μm^2^; 34.7 ± 2.4 × 10^3^ μm^2^ and 31.8 ± 1.7 × 10^3^ μm^2^, respectively).

### Vorinostat and olaparib enhanced cytotoxicity induced by standard chemotherapy drugs

**Figure 4:**
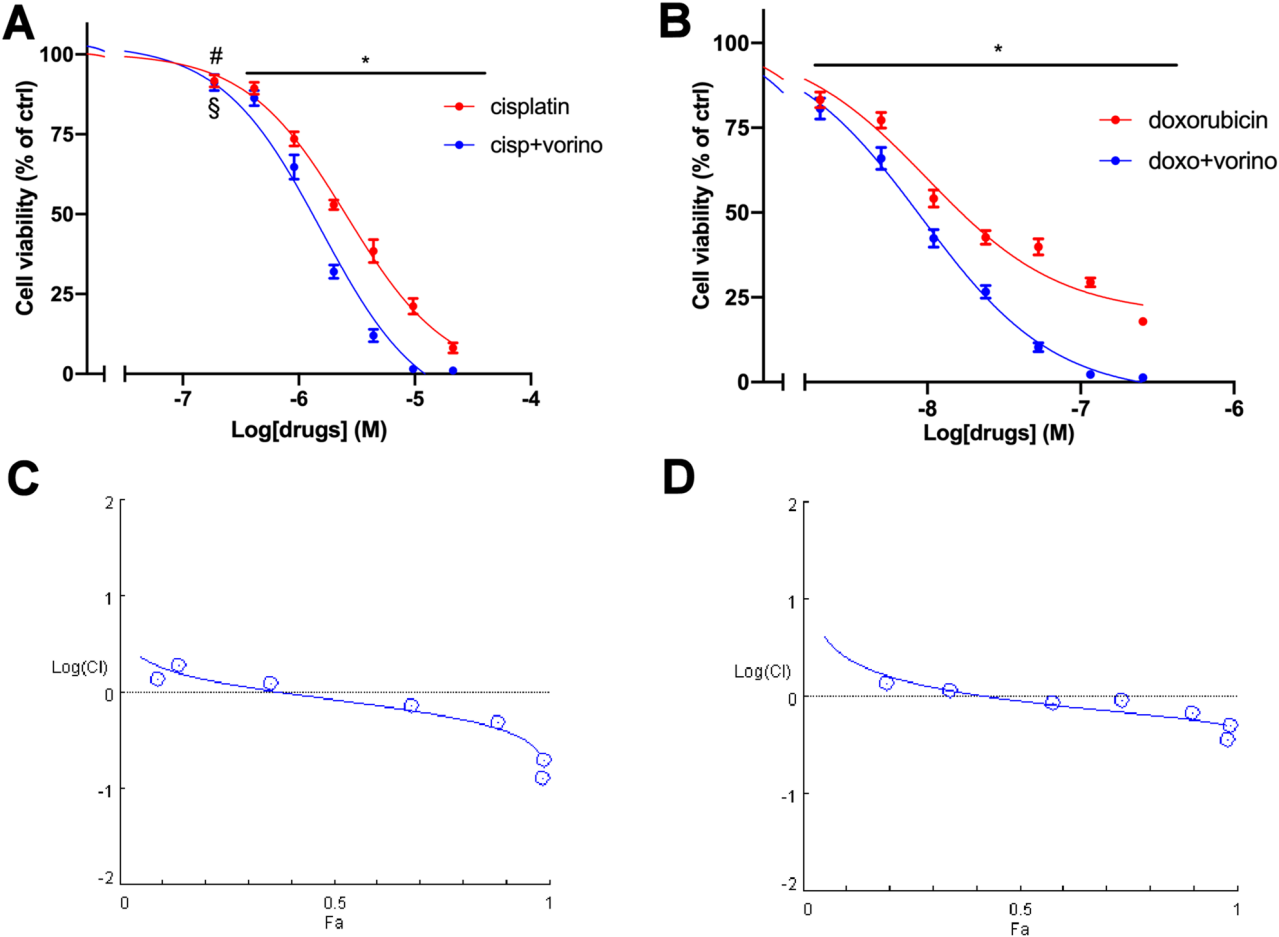
Vorinostat enhanced the effect of the chemotherapy drugs on hTERT cell viability. (**A**) Concentration–response curves of cisplatin− and cisplatin plus vorinostat-induced inhibition of cell viability. Cells were treated with increasing concentrations of cisplatin alone or in combination with vorinostat at fixed concentration molar ratio (cisplatin:vorinostat = 2.9:1) for 4 days. (**B**) Concentration–response curves of doxorubicin− and doxorubicin plus vorinostat-induced inhibition of cell viability. Cells were treated with increasing concentrations of doxorubicin alone or in combination with vorinostat at fixed concentration molar ratio (doxorubicin: vorinostat = 1:62.7) for 4 days. Cell viability was evaluated by MTT assay. Data are shown as mean ± SEM. (**C,D**) Semilogarithmic-Combination Index Plot of combined treatments with cisplatin plus vorinostat and doxorubicin plus vorinostat respectively. Dose and effect data obtained were converted to Fa values and analyzed with CompuSyn software [*p < 0.0001; ^#^p < 0.01; ^§^p < 0.05].

In order to evaluate the effect on ACC cells of target therapy drugs combined with either doxorubicin or cisplatin, the Chou–Talalay method was applied, as described in “Methods” section. The most promising results were obtained with the combination vorinostat plus cisplatin and vorinostat plus doxorubicin. In these cases, the combined treatments in ACC cells induced synergistic cytotoxic effects compared to each single compound. The concentration–response curves of single standard chemotherapy drug and of the combined treatments are reported in Fig. 4A,B. An increase in the potency was also observed in both combinations. Results obtained were analyzed with CompuSyn software; the combination index plots and the synergic effects are shown in Fig. 4C,D. The combination index (CI) values were < 1 starting from 0.68 to 2 μM for vorinostat and cisplatin, respectively. In the case of vorinostat/doxorubicin, CI < 1 was observed with 0.68 μM vorinostat and 0.011 μM doxorubicin.

**Figure 5:**
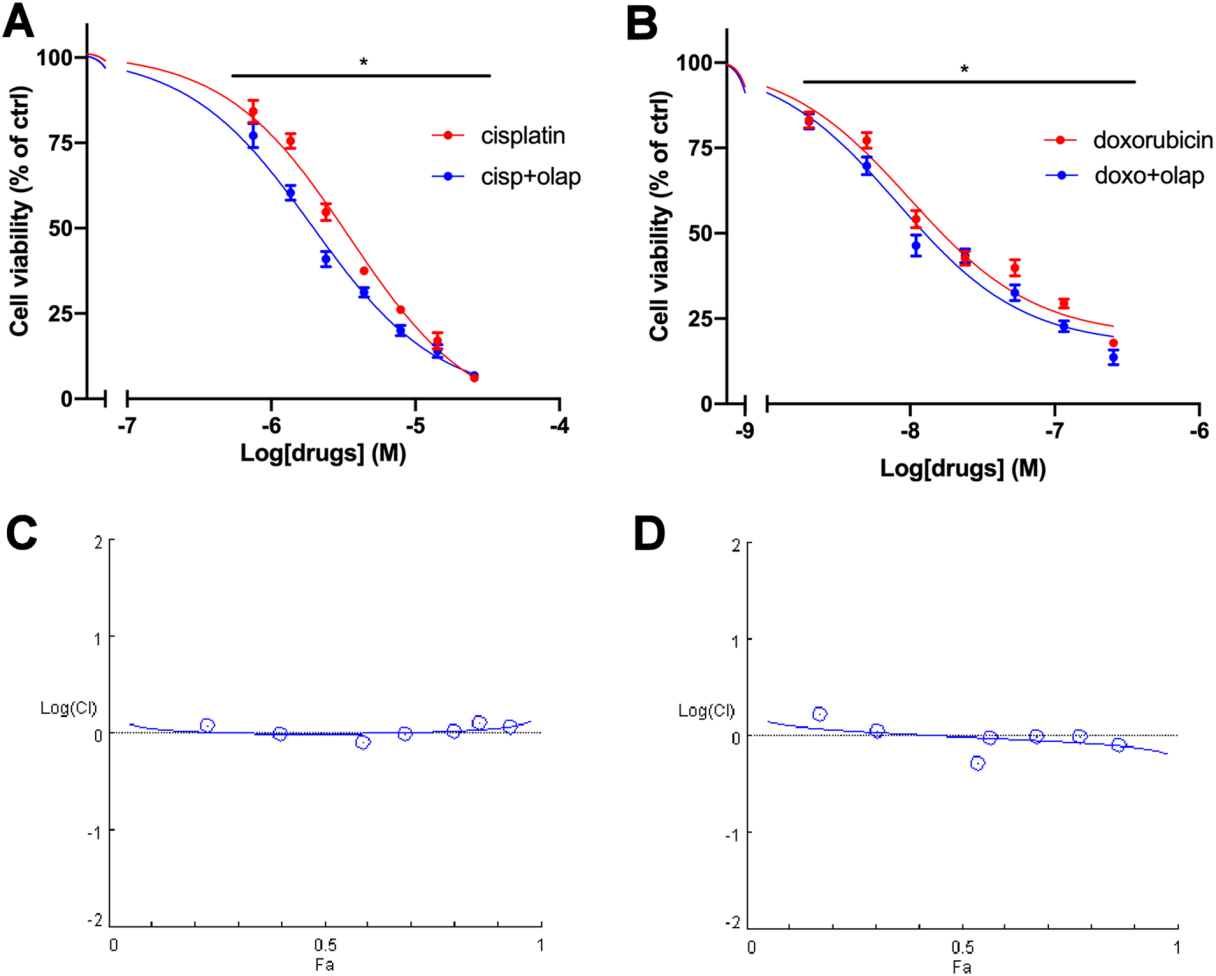
Olaparib enhanced the effect of the chemotherapy drugs on hTERT cell viability. (**A**) Concentration–response curves of cisplatin− and cisplatin plus olaparib-induced inhibition of cell viability. Cells were treated with increasing concentrations of cisplatin alone or in combination with olaparib at fixed concentration molar ratio (cisplatin:olaparib = 1:1.2) for 4 days. (**B**) Concentration–response curves of doxorubicin− and doxorubicin plus olaparib-induced inhibition of cell viability. Cells were treated with increasing concentrations of doxorubicin alone or in combination with olaparib at fixed concentration molar ratio (doxorubicin:olaparib = 1:104.3) for 4 days. Cell viability was evaluated by MTT assay. Data are shown as mean ± SEM. (**C,D**) Semilogarithmic-Combination Index Plot of combined treatments with cisplatin plus olaparib and doxorubicin plus olaparib respectively. Dose and effect data obtained were converted to Fa values and analyzed with CompuSyn software [*p < 0.0001].

Interestingly, olaparib treatment in ACC cells enhanced cytotoxicity induced by standard chemotherapy drugs. Indeed, both olaparib/cisplatin and olaparib/doxorubicin combined treatment increased cytotoxic effect as compared to each single-drug treatment which resulted in an increase in the potency (Fig. 5A,B). The Chou-Thalalay analysis confirmed the additive effect of the combined treatments (CI = 1, see Fig. 5C,D).

**Figure 6:**
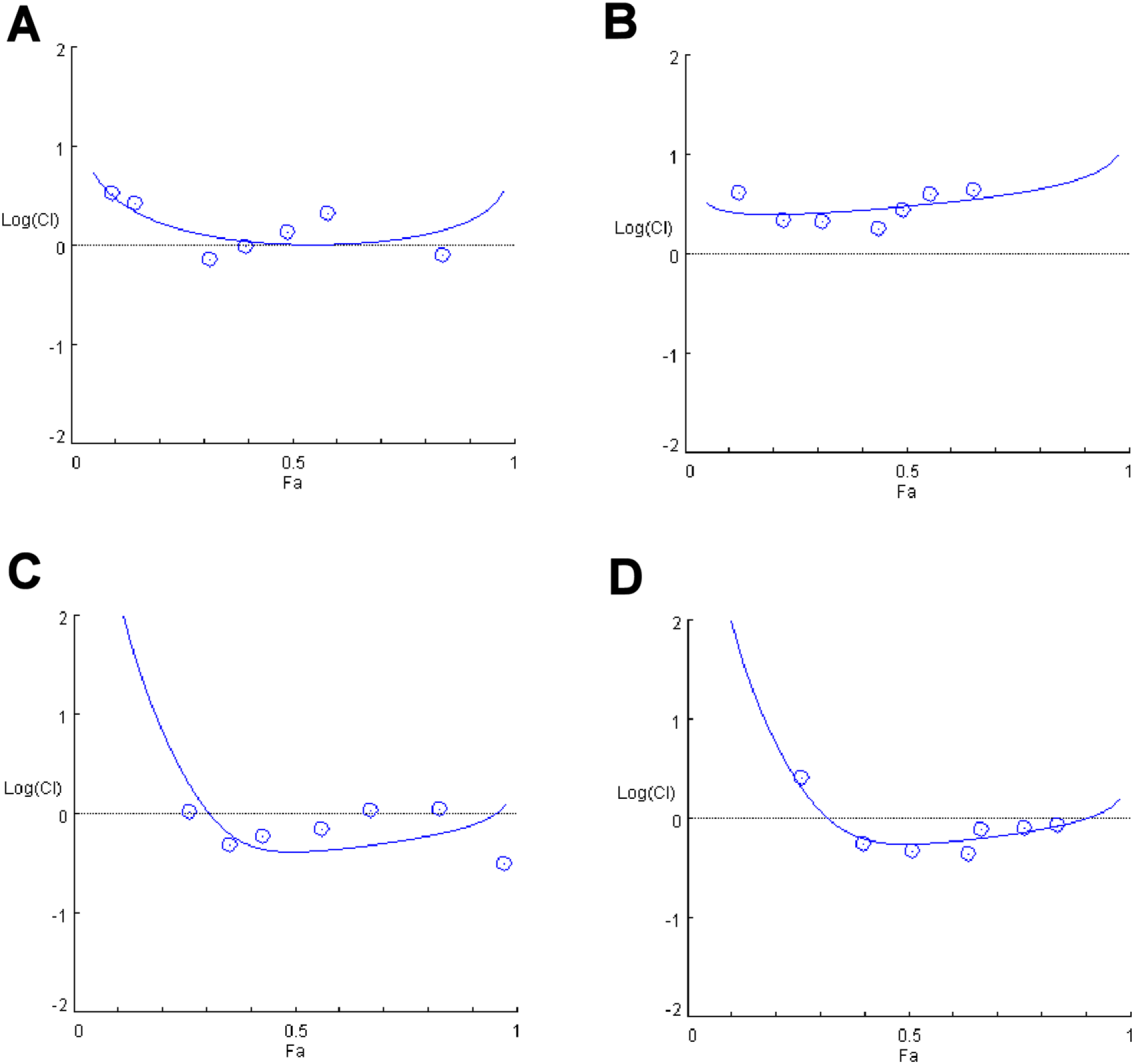
Effect of palbociclib and everolimus combined with chemotherapy drug. Semilogarithmic-Combination Index Plot of combined treatments with (**A**) cisplatin plus palbociclib, (**B**) doxorubicin plus palbociclib, (**C**) cisplatin plus everolimus and (D) doxorubicin plus everolimus. Dose and effect data obtained were converted to Fa values and analyzed with CompuSyn software.

All target therapy drugs were tested in combination with standard chemotherapy drugs, but as emerged in the analysis (Fig. 6), palbociclib and everolimus did not produce any significant increase in the single standard chemotherapy treatments. Specific concentration–response curves are reported in Fig. S4.

## Discussion

Chemotherapy has been for years the mainstay of treatment of relapsed ACC not eligible to locoregional treatment, but with no proofs of benefit in increasing survival^21–23^. No treatment options are present up to now for second line therapy^24^. Target therapy has emerged as a treatment option, but data till now available are quite disappointing. Single agents cetuximab, imatinib, dovitinib, bortezomib, cabozantinib, and bortezomib showed limited response rate (0–6%)^24^. Even combination therapy of chemotherapy plus imatinib or bortezomib did not show synergistic effects in unselected population^25, 26^. These unsatisfactory results underline the importance of comprehensively dissecting the therapeutic pathways involved in ACC and widely testing in preclinical models the activity of drug(s). The burden of mutations in ACC is low, with a few alterations recognized as therapeutically actionable^13^. This is mirrored by the paucity of trials aimed at delivering targeted treatments associated with known mutations or with altered molecular pathways. Moreover, the additive or synergistic effect of chemotherapy and targeted agents have not been widely studied at preclinical level.

ACC is a rare tumor and one of the main limitations for the research activity is the lack of models for the study of the disease. As an in vitro model we used the only cell line available, a human ACC cell line immortalized using h-TERT transfection. It has already been authenticated and well characterized^15^ and based on its genomic/proteomic profile (e.g., negative staining for p63) it resembles the more aggressive forms of ACC (ACC-I)^27^.

In the present study, human ACC (hTERT) cell line was used to test in vitro the pharmacological effects of standard chemotherapy and target therapy agents used in monotherapy or in combination, to give the preclinical bases for a pharmacological strategy for ACC treatment. We selected the most employed chemotherapy agents in ACC, cisplatin and doxorubicin^26^, studied to verify their cytotoxic and antiproliferative effect in the ACC experimental model used, and five target therapy agents, vorinostat, olaparib, palbociclib, everolimus and lenvatinib. The drug concentrations used in the study are consistent with the doses actually used in the clinic and with the resulted plasmatic concentrations^28–35^.

The rationale underlying the target therapy drugs chosen to evaluate their cytotoxic activity lies on data in literature, showing that the targets of these drugs were involved in the ACC carcinogenesis and progression. Indeed, it has been shown that recurring mutations in chromatin remodelling regulations genes are involved in ACC^36^. Vorinostat, a histone deacetylase inhibitor, showed response just in 7% of the case as monotherapy, but exerted its synergistic effect with cisplatin in preclinical models^37, 38^. Moreover, vorinostat in association with cisplatin demonstrated efficacy in depleting cancer stem cells and reducing tumor viability in ACC primary cell^38^. The PARP inhibitor olaparib was chosen because of the demonstrated presence in ACC of molecular alterations involved in DNA repair pathways, and thus possibly having a role both as single agent and in combination with chemotherapy^13^. Role of cycline dependent kinase (CDK) inhibitors has been recently evaluated in an in vitro study in ACC, with evidence of synergistic antitumoral activity in combination with cisplatin^39^. Everolimus may play a role in ACC, as, in an ACC cell line, Younes et al.^40^ showed the role of phosphatidylinositol 3-kinase (PI3K)-Akt-mammalian target of rapamycin (mTOR) as potentially target of therapy. Everolimus in a phase 2 study^41^ showed a median progression-free survival (PFS) of 11.2 months and about 80% of stable disease, however no partial or complete response were achieved. Finally, lenvatinib is a second-generation multiple kinase inhibitor with a strong anti-angiogenic effect; it reported responses in the range of 12–16% and high rate of disease stabilization (about 70% of patients), but with half of the patients reporting toxicity of grade 3 or greater^11, 12^.

Our results demonstrated the in vitro cytotoxic activity of the standard chemotherapy drugs. Among targeted agents, vorinostat reported the highest efficacy, with a cell mortality of over 90%, while modest efficacy emerged for olaparib and palbociclib and poor efficacy for everolimus. Finally, lenvatinib did not induce significant cytotoxic effects in ACC (hTERT) cell line, and this poor performance could be explained by the lack of vascular components of in vitro models, that are indeed one of the main mechanisms of action of this drug. The in vitro cytotoxic effect of all the drugs tested was confirmed and strengthen by results obtained in the in vivo model of ACC (hTERT) cell line xenograft in zebrafish embryos. This animal model offers a valid and useful tool for in vivo first drug screening^20, 42^.

In the context of ACC tumor, several studies confirmed that a two-drug polychemotherapy gives better response rate compared with monotherapy^43^, even if with limited duration and uncertain benefit in overall survival. Therefore, the association of chemotherapy and targeted agents is a logical step to be carried out^44^ and we tried to demonstrate the activity of these combinations in the in vitro model of ACC cell line. Our results demonstrated that the two standard chemotherapy agents (cisplatin or doxorubicin) plus vorinostat or olaparib were effective. Indeed, vorinostat significantly increased both the efficacy and the potency of the standard chemotherapy agents and the combination exerted a synergic effect. Also olaparib improved the performance of the two chemotherapy agents, resulting in an addictive effect. These effects found their rationale in the specific molecular mechanism of the drugs: both cisplatin and doxorubicin hamper DNA replication and RNA transcription, the first inducing the formation of intra- and inter-strand cross-link of nuclear DNA^45^, the second interacting with DNA by intercalation and inhibiting macromolecular biosynthesis. This effect finally leads to cell cycle arrest and apoptosis. Vorinostat increases the formation of hyperacetylated histones, which therefore blocks the interaction of histone with DNA and leaves it more accessible by cisplatin or doxorubicin. Therefore, the synergistic effect induced by vorinostat may be due to the action upstream of the chemotherapy agents, which facilitates and greatly enhances their effects and this result in ACC cells is in line with the finding that vorinostat sensitizes ACC tumor cells to chemotherapy^38^. Olaparib, on the other hand, preventing the repair of the DNA damages induced by platinum compounds or doxorubicin^46^, acts downstream of cisplatin and doxorubicin, adding a further barrier to cell proliferation.

Taken together, our results indicate that combined treatment with vorinostat or olaparib with standard chemotherapy agents cisplatin and doxorubicin is significantly more effective than monotherapy. These data set the basis for further studies in a dedicated prospective clinical trial. The drug combination proposed have already been examined in clinical trials. Back in 2014, a phase I trial showed tolerability and promising activity of the combination PARPi + cisplatin in solid tumors^47^. Given the strong preclinical rationale of synergistic effects between CDK inhibitors and platinum-based therapy^48^ combination is under evaluation in an ongoing phase I clinical trial (NCT02897375). Finally, phase I-II study on HDAC inhibitors vorinostat with platinum chemotherapy agents showed promising clinical activity and good tolerability, even in a triplet combination with taxol or capecitabine^49, 50^.

## Supplementary Information

Supplementary Figures.

## Data Availability

All data generated or analysed during this study are included in this published article [and its Supplementary Information files]. Further details are available on request from the corresponding author.
