# Supplementary material for: Cytotoxic effects of targeted agent alone or with chemotherapy in the treatment of adenoid cystic carcinoma: a preclinical study
Source: Sci Rep. 2022 Jun 15;12:9951. doi: 10.1038/s41598-022-14197-8 (PMC9200834; doi:10.1038/s41598-022-14197-8)
Supplement: Supplementary file 1 — Supplementary Figures. [file 41598_2022_14197_MOESM1_ESM.pdf]

Supporting information

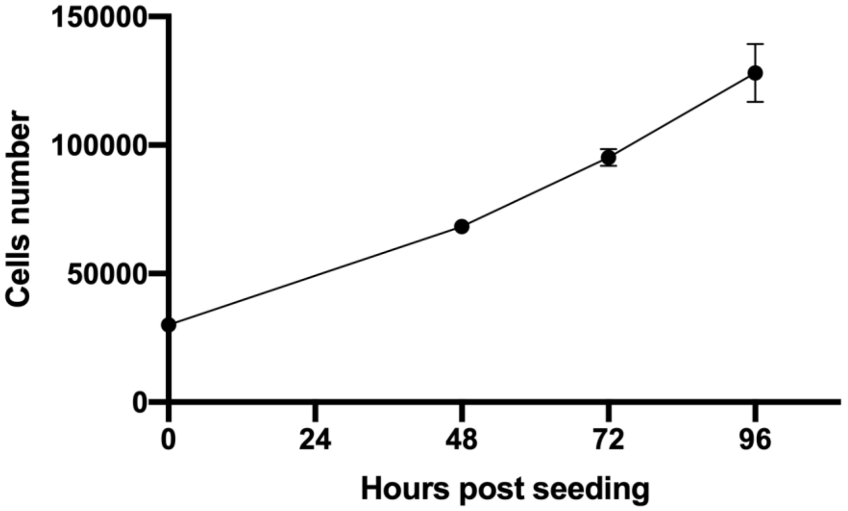

S1 Fig. hTERT cells growth curve at 37°C. ~30.000 cells were seeded in each well and cultured at 37°C, 5% CO_2. Cells proliferation was evaluated after 48, 72 and 96 hours by cells count with trypan blue exclusion. The doubling-time was evaluated with the following formula: DT=T ln2/ln(Xe/Xb), where T is the incubation time in any units, Xb is the cell number at the beginning of the incubation time, Xe is the cell number at the end of the incubation time

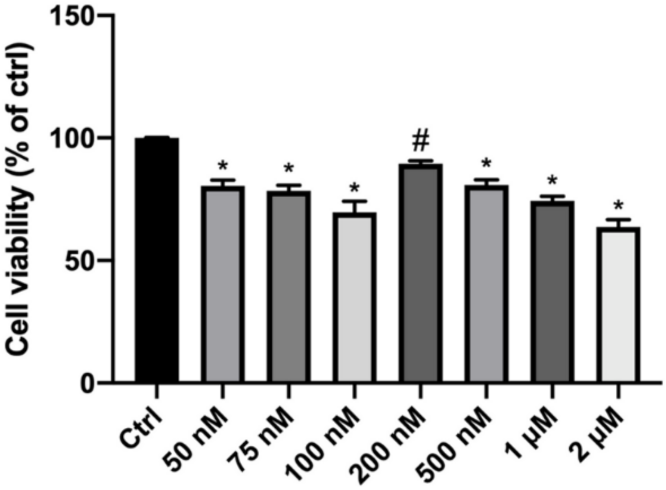

S2 Fig. Lenvatinib had little impact on hTERT cell viability. Cells were treated with increasing concentrations of lenvatinib (0.05-2 μM) for 4 days. Cell viability was evaluated by MTT assay. Data are shown as mean ± SEM. [* $p < 0.0001$; # $p < 0.01$]

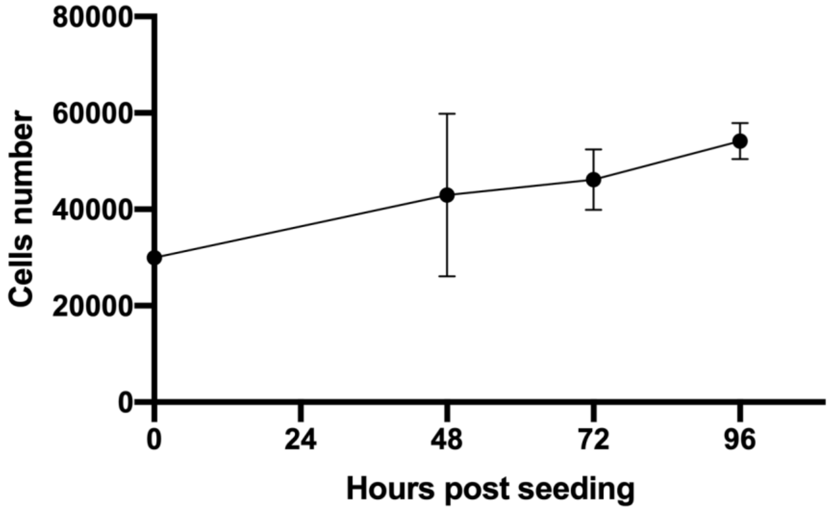

S3 Fig. hTERT cells growth curve at 32°C. ~30.000 cells were seeded in each well and cultured at 32°C, 5% CO_2. Cells proliferation was evaluated after 48, 72 and 96 hours by cells count with trypan blue exclusion. The doubling-time was evaluated with the following formula: DT=T ln2/ln(Xe/Xb), where T is the incubation time in any units, Xb is the cell number at the beginning of the incubation time, Xe is the cell number at the end of the incubation time

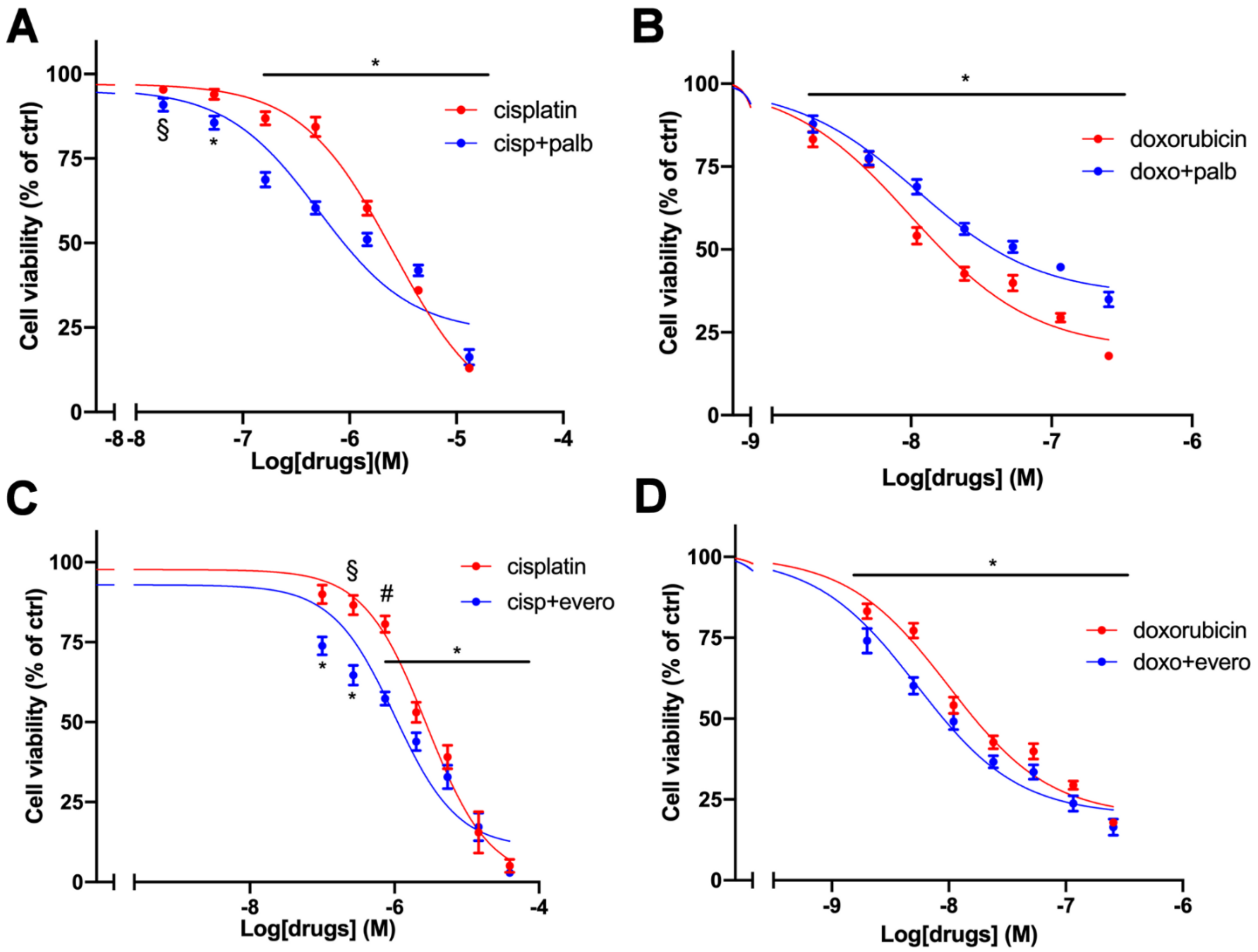

S4 Fig. Effect of palbociclib and everolimus combined with chemotherapy drug. (A) Concentration-response curves of cisplatin- and cisplatin plus palbociclib-induced inhibition of cell viability. Cells were treated with increasing concentrations of cisplatin and palbociclib alone or in combination at fixed concentration molar ratio (cisplatin : palbociclib = 1 : 1.1) for 4 days. (B) Concentration-response curves of doxorubicin- and doxorubicin plus palbociclib-induced inhibition of cell viability. Cells were treated with increasing concentrations of doxorubicin and palbociclib alone or in combination at fixed concentration molar ratio (doxorubicin : palbociclib = 1 : 11.8) for 4 days. (C) Concentration-response curves of cisplatin- and cisplatin plus everolimus-induced inhibition of cell viability. Cells were treated with increasing concentrations of cisplatin and everolimus alone or in combination at fixed concentration molar ratio (cisplatin : everolimus = 250 : 1) for 4 days. (D) Concentration-response curves of doxorubicin and doxorubicin plus everolimus-induced inhibition of cell viability. Cells were treated with increasing concentrations of doxorubicin

and everolimus alone or in combination at fixed concentration molar ratio (doxorubicin : everolimus = 3.6 : 1) for 4 days. Cell viability was evaluated by MTT assay. [* $p < 0.0001$; # $p < 0.01$; § $p < 0.05$]
